# Exploring the Interactions between two Ligands, UCB-J and UCB-F, and Synaptic Vesicle Glycoprotein 2 Isoforms

**DOI:** 10.1021/acschemneuro.4c00029

**Published:** 2024-05-03

**Authors:** Junhao Li, Rongfeng Zou, Andrea Varrone, Sangram Nag, Christer Halldin, Hans Ågren

**Affiliations:** † Department of Physics and Astronomy, Uppsala University, Box 516, Uppsala SE-751 20, Sweden; ‡ Department of Clinical Neuroscience, Centre for Psychiatry Research, Karolinska Institutet and Stockholm Health Care Services, Stockholm 171 77, Sweden

**Keywords:** synaptic vesicle glycoprotein 2, in silico modeling, molecular dynamics simulations, positron emission tomography

## Abstract

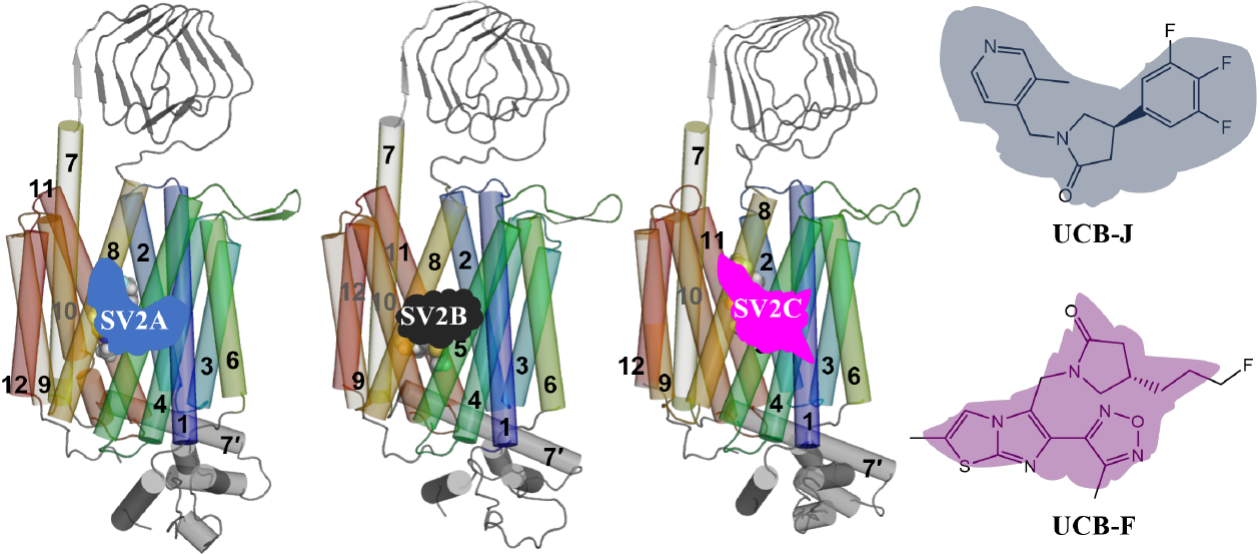

*In silico* modeling was applied to study the efficiency of two ligands, namely, **UCB-J** and **UCB-F**, to bind to isoforms of the synaptic vesicle glycoprotein 2 (SV2) that are involved in the regulation of synaptic function in the nerve terminals, with the ultimate goal to understand the selectivity of the interaction between **UCB-J** and **UCB-F** to different isoforms of SV2. Docking and large-scale molecular dynamics simulations were carried out to unravel various binding patterns, types of interactions, and binding free energies, covering hydrogen bonding and nonspecific hydrophobic interactions, water bridge, π–π, and cation−π interactions. The overall preference for bonding types of **UCB-J** and **UCB-F** with particular residues in the protein pockets can be disclosed in detail. A unique interaction fingerprint, namely, hydrogen bonding with additional cation−π interaction with the pyridine moiety of **UCB-J**, could be established as an explanation for its high selectivity over the SV2 isoform A (SV2A). Other molecular details, primarily referring to the presence of π–π interactions and hydrogen bonding, could also be analyzed as sources of selectivity of the **UCB-F** tracer for the three isoforms. The simulations provide atomic details to support future development of new selective tracers targeting synaptic vesicle glycoproteins and their associated diseases.

## Introduction

**Figure 1:**
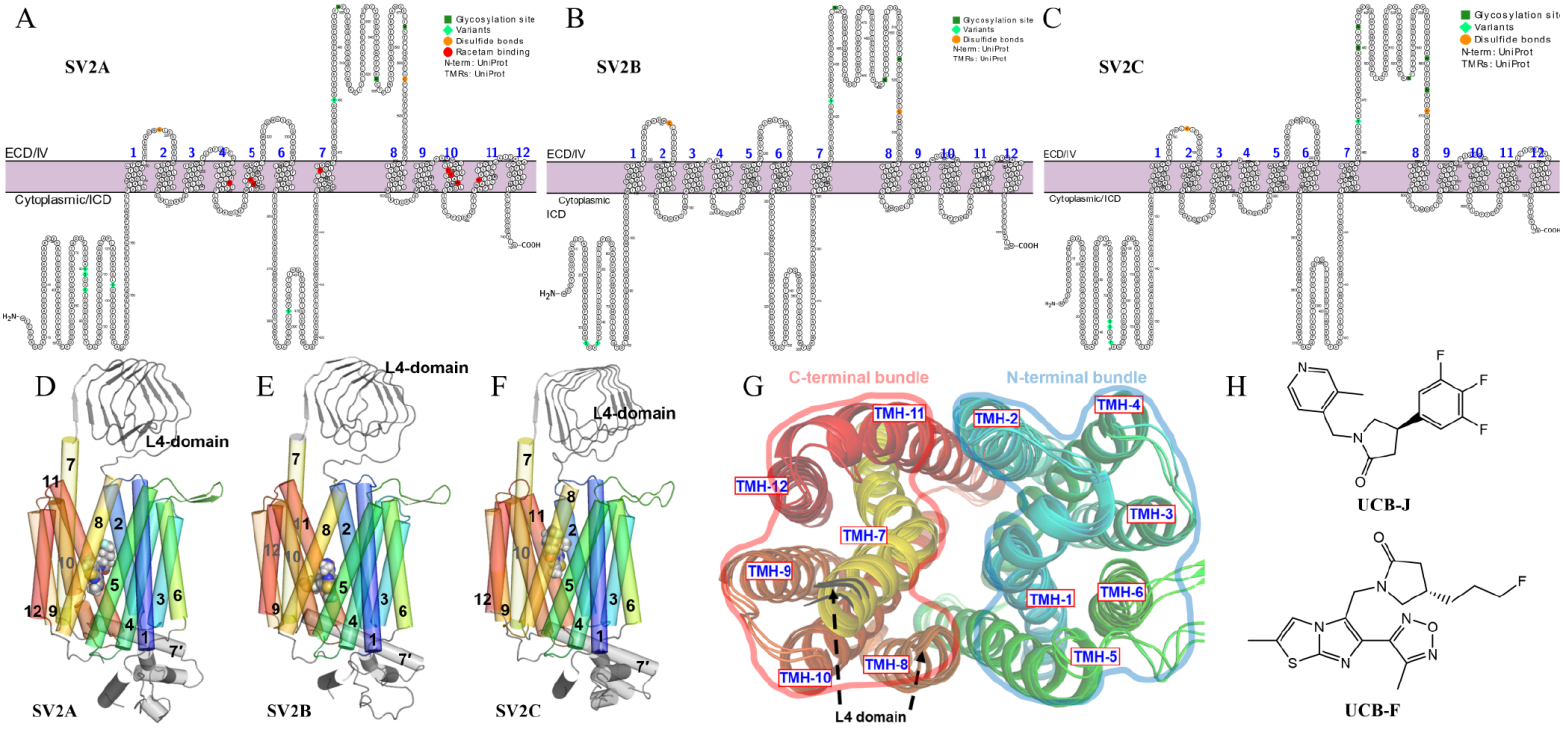
Sequences and structures of SV2. (A–C) Full sequences of SV2A, SV2B, and SV2C with glycosylation site (green), variants (light green), disulfide sites (orange), and racetam drugs binding sites (red) annotated. ECD: extracellular domain, IV: intra vesicular, ICD: intracellular domain. Transmembrane helices (TMHs) are labeled with blue bold numbers. The figures were manually generated using the protter web server (http://wlab.ethz.ch/protter/start/).^35^ (D–F) Front view of the entire SV2 proteins. **UCB-F** and the helices are rendered as spheres and cylindrical shapes, respectively, with the numbering of transmembrane helices labeled. (G) Top view of the TMHs from the ECD side for the superimposed representative models of SV2A, SV2B, and SV2C from the RoseTTAFold predictions.^34^ Other regions of the protein are not displayed. (H) The structures of **UCB-J** and **UCB-F**.

The synaptic vesicle glycoprotein 2 (SV2) family of proteins are involved in the regulation of synaptic function in the nerve terminals.^1−6^ A majority of them are ubiquitously distributed in the central nervous system (CNS).^2^ Similar to the members of the solute carrier (SLC) family (or major facilitator family), the SV2 family forms transmembrane proteins that have 12 transmembrane helices (TMHs) with the *N*- and C-termini located in the cytoplasmic side (Figure 1A–C).^7^ Although the topologies of their secondary structures are similar to those of transporter proteins, the exact functions of the SV2 protein remain unclear.^5^ Despite their functions, SV2 proteins are putatively characterized as a special subfamily in the SLC family (https://slc.bioparadigms.org/).^8^ The members in the SV2 family share about 60% identity in their sequences, of which the TMH areas are more identical.

Three isoforms of the family have been characterized for vertebrates, coined as SV2A, SV2B, and SV2C.^9−11^ SV2A is extensively distributed throughout the entire CNS; SV2B is also widely expressed in the brain except the regions of subtantia nigra, globus pallidus, dentate gyrus, and the reticular nucleus of the thalamus, whereas expressions of SV2C are much more limited and are only found in the pallidum, brainstem, striatum, and substantia nigra.^9−13^ Although synaptic vesicles are often demoted as neurotransmitter vesicles and SV2 was initially assumed to be a novel transporter for neurotransmitters,^3^ there is no direct evidence supporting such transportation mediated by SV2 proteins. Interestingly, the luminal domain (Figure 1D–F) between TMHs 7 and 8 of SV2 has been found to contain the glycosylation sites and acts as receptors for the binding of botulinum and tetanus neurotoxins, suggesting its potential role in the transportation of neurotoxin proteins.^14−16^ Also, whether the luminal glycosylation plays a role in the binding of small molecules to SV2 protein remains elusive.

Considering its ubiquitous expression in the brain, SV2A imaging with positron emission tomography (PET) has been used as an important tool to study synaptic density in different neuropsychiatric disoders.^17−21^ On the basis of being the binding receptor of the antiepileptic drug levetiracetam discovered by Lynch et al.,^22^ a series of compounds containing the 2-pyrrolidone core have been developed as PET tracers for imaging SV2A *in vivo*.^19,23−26^ Initially, the affinity of levetiracetam to SV2A (*K*_i_ = 2.5 μM) was found to be too low for *in vivo* PET applications.^17^ To improve the affinity, the 1-acetamide of levetiracetam was replaced by larger functional groups containing an aromatic ring, followed by the substitution of the *pro-4R-*hydrogen of the 2-pyrrolidone core with another aromatic functional group. Among these compounds, **UCB-J** (Figure 1H) was found to have high affinity and selectivity (p*K*_i_ = 8.15, or *K*_i_ = 7 nM) to SV2A and suitable pharmacokinetics properties for *in vivo* PET imaging.^24^

Inspired by the selective binding of levetiracetam to SV2A, researchers in UCB pharma disclosed a new selective SV2C binder (named **UCB-F**, Figure 1H) a decade ago.^27^ Similar to the series of selective SV2A compounds, the substitution of the 2-pyrrolidone core took place at the 1- and *pro-4R*-positions. However, the pharmacokinetics in rat brain appears unfavorable for the use of [^18^F]**UCB-F** to quantify SV2C *in vivo.* Dunn et al. have reported that SV2C is selectively enriched in the basal ganglia, is disrupted in animal models of PD, and is involved in the modulation of dopamine release.^28−31^ Therefore, PET tracers for imaging SV2C could be developed as potential biomarkers for Parkinson’s disease.

The *in vitro* mutagenesis study performed by Shi et al. confirmed that the racetams bind to the TMH domain of SV2A.^32^ However, not all the residues aligned to the key substrate recognizing residues in the SLC family exhibit altered binding of **UCB30889** upon alanine scanning.^32,33^ Considering the high sequence identities among the SV2 proteins, one can assume that the TMH domain in SV2B and SV2C also serves as binding sites of small molecules, for example, for PET tracers. Superimposing the high quality deep-learning predicted structures of SV2 proteins also indicates the high similarity of their TMHs in both sequences and structures (Figure 1G).^34^ In this work, we capitalize on the development of *in silico* simulation methods in order to study the microscopic mechanisms of ligand binding and to disclose key binding patterns that in turn lay the grounds for their further optimization with improved performance. Starting from the structures reviewed above, we have performed long-time molecular dynamics (MD) simulations to study the structural dynamics and stabilities of the three SV2 proteins, followed by the induced-fit docking of **UCB-J** and **UCB-F** into these models and long-time MD simulations of each complex. The dynamic interactions between **UCB-J** and **UCB-F** and SV2 proteins are dissected by the simulation interaction diagram using the trajectories from different periods of the simulations. Finally, the binding free energies of **UCB-J** and **UCB-F** were also calculated and compared to their selectivity preference.

## Results and Discussion

### Structure Models of SV2

The sequences of SV2 (isoforms A–C) share ∼60% identities (Figure S1). Of the 12 TMHs, some residues on TMHs 4, 5, 7, 10, and 11 are important to the binding of racetam drugs from *in vitro* mutation experiments.^32,33^ The representative models from the RobettaFold prediction clearly show a tunnel that might be responsible for transportation of small molecules (Figure 1G). Thereby, the TMHs are separated into two subdomains, comprising TMHs 1–6 and 7–12, respectively (Figure 1G). Superimposition of all the top-5 ranked models indicate that the positions of the N-terminal helix are highly flexible and uncertain (Figure S2). The position of the N-terminal helix is also highly uncertain in the top-5 ranked AlphaFold2 predictions,^36^ for which the prediction confidence is very low. We have ruled out the structures with the predicted N-terminal helix buried in the membrane area (Figure S2).

**Figure 2:**
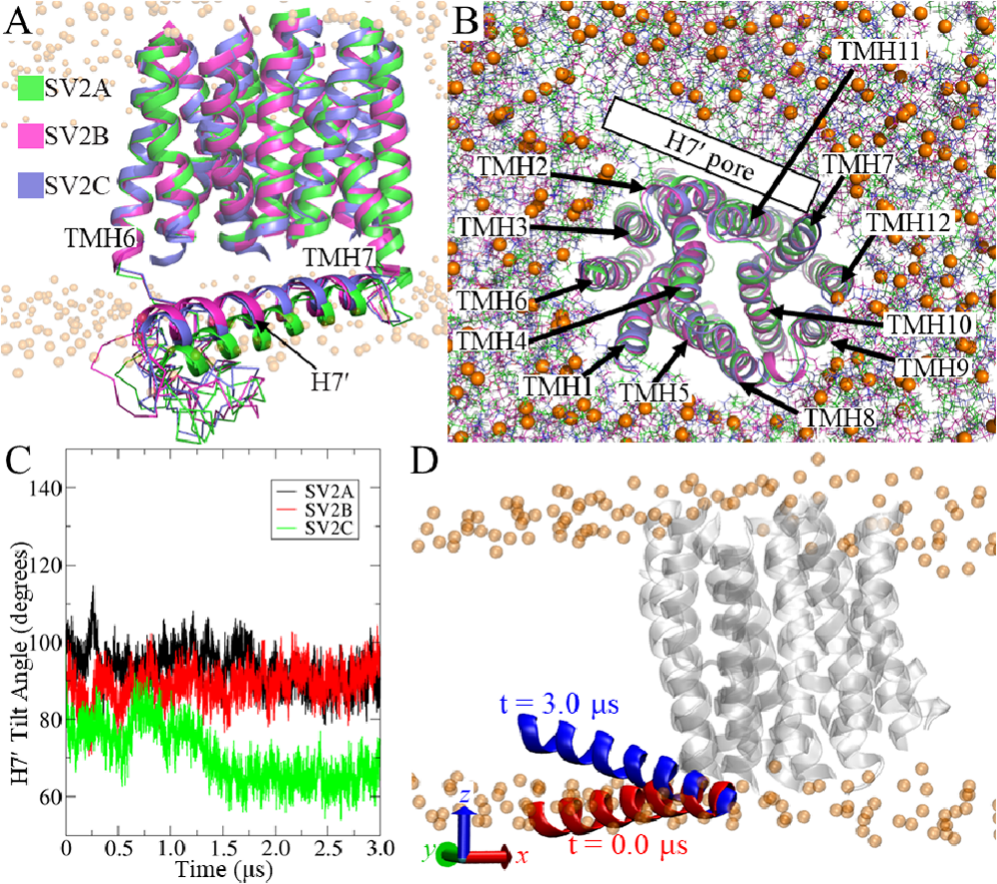
Conformation of helix neighboring TMH7 (named H7′). (A) Comparison of the H7′ in the initial models. (B) An additional pore on the ICD side of the membrane due to the orientation of H7′. (C) Time course evaluation of the H7′ tilt angle (cross with the *z*-axis). (D) Conformation of H7′ from *t = 0.0* and *t = 3.0* μs in the SV2C simulations. The phosphate atoms and the other atoms of the membrane are depicted as orange spheres and lines, respectively.

The long-length domain in ICD connecting the TMH6 and TMH7 is predicted to be dominated by helices, one of which, namely, H7′, is neighboring TMH7 and lays on the membrane interface (Figure 2A). Short-time (less than 1 μs) MD simulations indicate that the position of this helix was maintained, leading to a pore on the phosphorylated interface (Figure 2B). We have analyzed the sequences of the H7′ helices (see the green box in Figure 1, length: 26 residues), and the net charges are +2, + 3, and +4 for SV2A, SV2B, and SV2C, respectively. We suppose that the position and positive net charges are important for the “laying-down” orientation of H7′, because the membrane interface is rich with negatively charged PO_4_^–^ moieties. Interestingly, the angles between H7′ and the membrane surface appear to follow the same order as the net charges (Figure 2A).

Considering that H7′ is connecting TMH7 that has a racetam binding residue (Figure 1A), we extended the simulations of the full-length structures to 3 μs. From the trajectories in 2.5–3.0 μs, we observed that the tilt angle (crossed with the *z*-axis) of H7′ decreased significantly for the SV2C model (Figure 2C), indicating that it was embedded into the membrane (Figure 2D). Since no experimental evidence shows that H7′ is a transmembrane helix, we have manually morphed H7′ to be closer to the cytoplasmic in the Maestro (version 2021-4) workspace (Figure 2). Also, the highly uncertain N-terminal helix was removed before simulations with **UCB-J** and **UCB-F**.

### Dynamical Interactions between UCB-J and SV2

**Figure 3:**
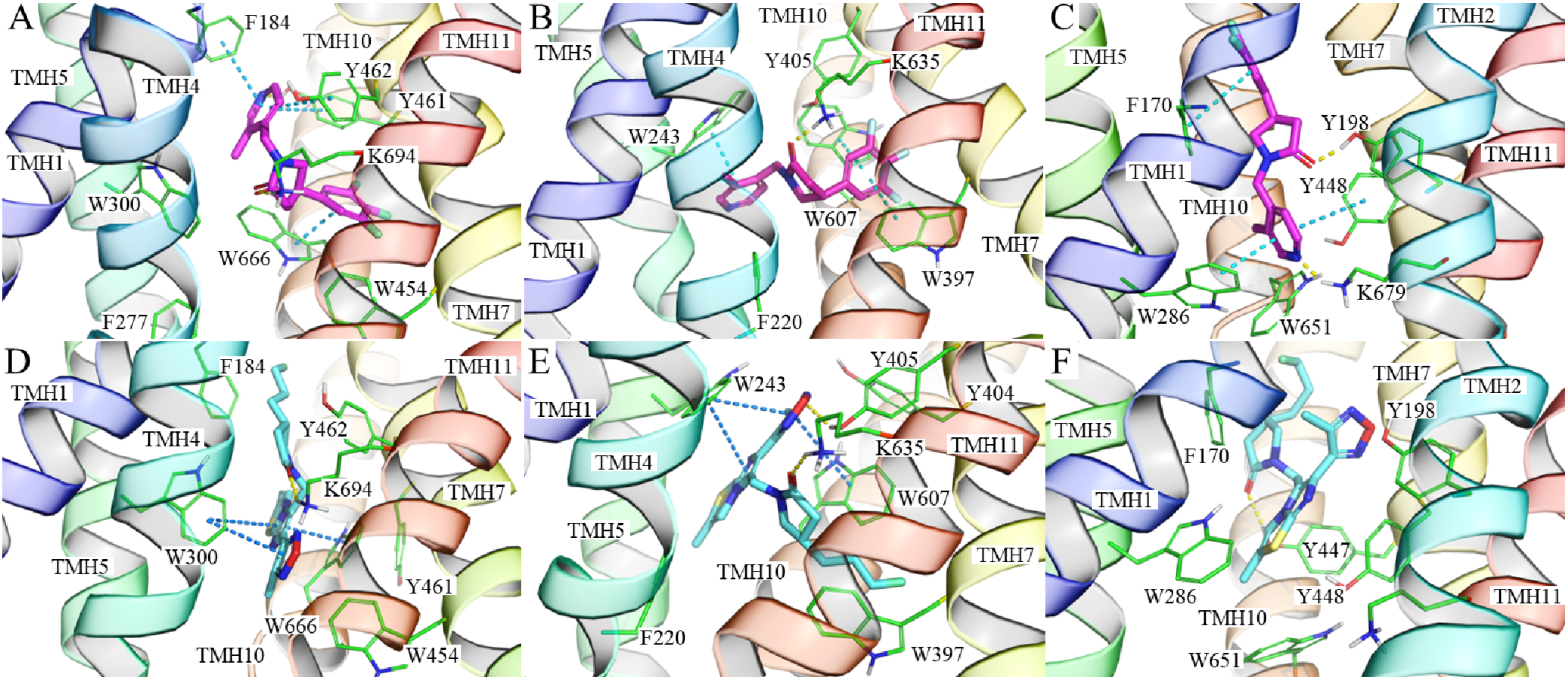
Initial binding modes from induced-fit docking. **UCB-J** (A–C) and **UCB-F** (D–F) are depicted as magenta and cyan sticks, respectively. The interacting residues are colored thin green sticks with the corresponding TMH labeled. (A) and (D) for SV2A, (B) and (E) for SV2B, and (C) and (F) for SV2C.

Preceding studies have indicated that W300^TM5^, Y462^TM7^, W666^TM10^, and K694^TM11^ are important in recognizing racetam drugs in SV2A.^32,33^ The aligned residues for W300^TM5^, Y462^TM7^, W666^TM10^, and K694^TM11^ in SV2B and SV2C are W243^TM5^, Y405^TM7^, W607^TM10^, and K635^TM11^ and W286^TM5^, Y448^TM7^, W651^TM10^ and K679^TM11^, respectively (Figure S1). Since knowledge of the distances between these residues is not sufficient to fit the extended functional groups (at the 1- and 4-positions of the pyrrolidone core) in **UCB-J** and **UCB-F,** the initial binding modes of **UCB-J** and **UCB-F** in the TMH cavities of SV2 were obtained by the induced fit docking module implemented in the Schrodinger suite (version 2021-4). The results indicate that the ligands in all the outputted poses adopted different orientations in the binding pocket defined by the above residues (Figure 3), and therefore, the top-1 ranked binding modes were selected for each system. The trifluorophenyl moiety of **UCB-J** is located in the second TMH bundles of SV2A and SV2B, exhibiting π–π interactions with W666^10^ and W397^10^ and W607^10^, respectively (Figure 3A,B). In SV2C, the orientation of **UCB-J** is more parallel to the *z*-axis than in SV2A and SV2B and exhibits more interactions, including hydrogen bonds with Y198^2^ and K679^11^ and π–π interactions with W286^5^, Y448^7^, and F170^1^ (Fgiure 3C). There are other orientations that are similar to **UCB-J** in SV2A and SV2B; however, they have lower ranking affinities and much fewer interactions with SV2C. The pyrrolidine-2-one moiety is the major functional group of racetam drugs, which is kept in the structures of **UCB-J** and **UCB-F**. In the induced-fit docking, this moiety in **UCB-J** was found to form hydrogen bonds with K694^11^, K635^11^, and Y198^2^ in SV2A, SV2B, and SV2C, respectively (Figure 3, parts A–C). Similar hydrogen bonding modes are also found in complexes bound with **UCB-F**, in which the oxygen of the pyrrolidine-2-one moiety is hydrogen bonding with K694^11^, K635^11^, and Y447^7^ in SV2A, SV2B, and SV2C, respectively (Figure 3D–F). Hydrophobic interactions were also found between ligands and 6 TMHs (3 from the N-terminal bundle and 3 from the C-terminal bundle). Despite the similar initial conformations (Figure 1G), we note that SV2C exhibits different preference for the interacting TMHs from N-terminal bundle (TMHs 1, 2, and 5), compared to SV2A and SV2B (TMHs 1, 4, and 5).

The complexes derived from docking were then subjected to 2000 ns MD simulations. The root-mean-square deviation (RMSD) values indicated that the backbone atoms in TMHs are much more stable than those in other regions (Figure S3). The RMSD values of ligands in all 6 systems lie mostly above 3 Å, especially for **UCB-J** in SV2C, which reaches 10 Å in the last ∼150 ns trajectory (Figure S3). By comparing the binding modes of **UCB-J** and **UCB-F** from the beginning and ending of trajectories, we found that **UCB-J** was dissociating from the binding site of SV2C in the last 150 ns simulations (Figure S3). Although the upright binding mode of **UCB-J** can form more hydrogen bonds with SV2C (Figure 3C), it has become horizontal to the upper membrane plane (Figure S4), suggesting that the binding pocket of SV2C cannot stabilize the pyridine ring moiety of **UCB-J**. In contrast, **UCB-J** in SV2A exhibits a trend to shift the lay-down bind mode to the upright mode. **UCB-F** in SV2C decreased after MD simulations, which became closer to the ICD side.

**Figure 4:**
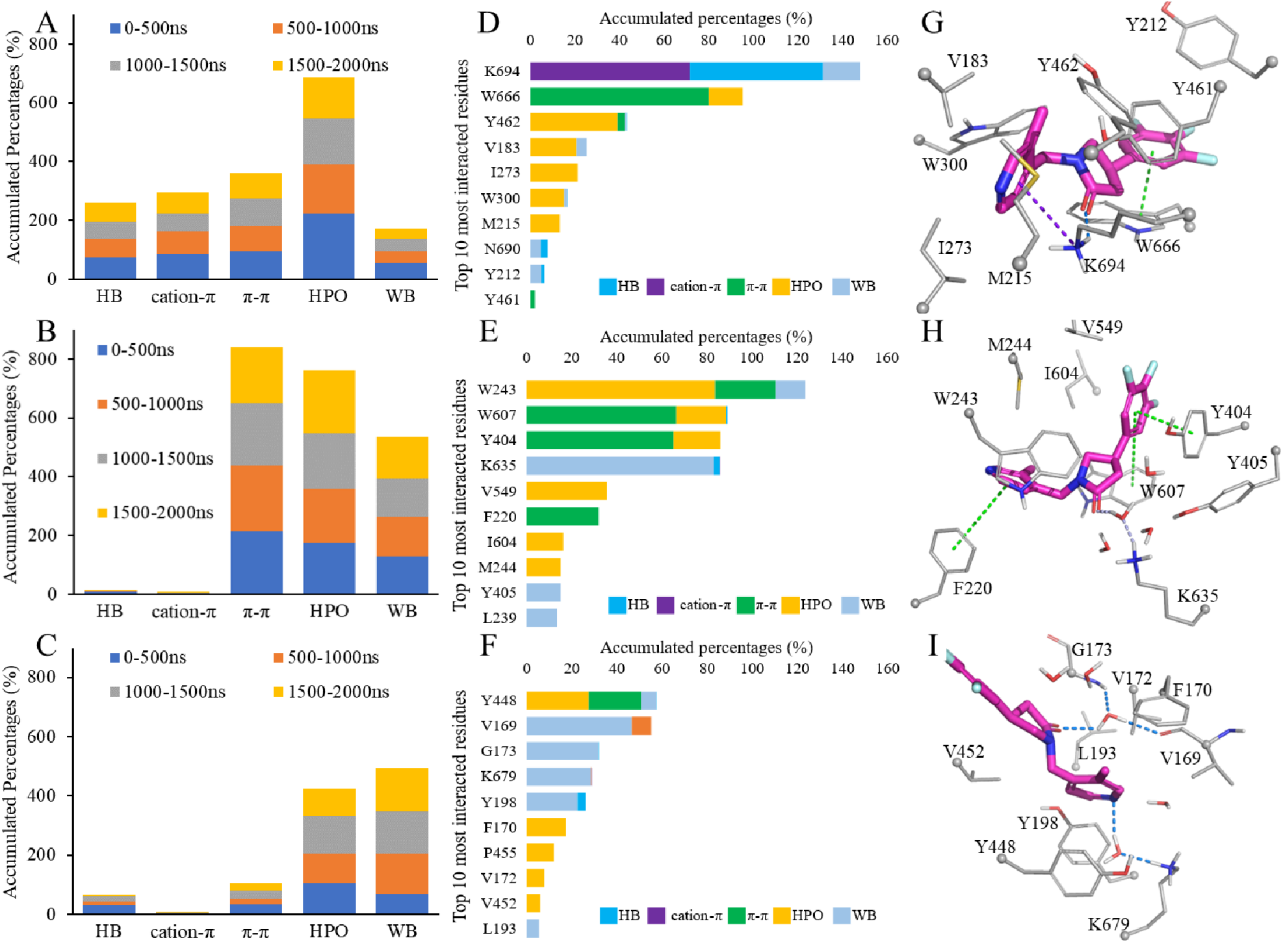
Simulation interaction diagrams for **UCB-J** in SV2A (subfigures A–G in the first row), SV2B (B–H in the second row), and SV2C (C–I in the third row). Hydrogen bonding, nonspecific hydrophobic, and water bridge interactions are abbreviated as HB, HPO, and WB, respectively. The counting of hydrophobic interactions excludes the π–π and cation−π interactions. The top-10 most interacting residues in the trajectories of 1500–2000 ns are dissected in parts D–F. The representative snapshots are presented in parts G–I with the key residues, Cα atoms, and **UCB-J** colored in gray thin sticks, small spheres, and magenta sticks, respectively.

We further performed simulation interaction analysis for all of the systems. From docking studies, the 2-one atom of the 2-pyrrolidone core is an important hydrogen bond receptor for interactions with SV2 proteins. This feature was well maintained throughout the MD simulations of **UCB-J** in SV2A, while there are only a few hydrogen bonding interactions for **UCB-J** in SV2B and SV2C (Figure 4A–C and Figure S5). For the specific hydrophobic contacts, the cation−π interaction only existed throughout ∼70% of the last 500 ns MD trajectory of **UCB-J** in SV2A, which was contributed by the amine terminal of K694 and the pyridine moiety of **UCB-J** (Figure 4D,G). Meanwhile, K694 is the major hydrogen bond donor to the 2-one oxygen atom of **UCB-J** in 60% of frames from the SV2A/**UCB-J** trajectory (1500–2000 ns). W666 is one of the important residues for racetam binding to SV2A, and it was found to form steady π–π interactions with the trifluorophenyl group of **UCB-J**. Contacts between **UCB-J** in SV2A with Y462 and W300 are mainly contributed by nonspecific hydrophobic interactions (Figure 4D). The other residues that is important for racetam binding to rat SV2A,^32^ such as F277, G303, F658, and V661, were not found in the top-10 most interacted residues throughout the last 500 ns trajectory (Figure 4D). For **UCB-J** in SV2B and SV2C, much more water bridges were found compared to that in SV2A, indicating that the direct contacts, such as hydrogen bonds, in the initial binding mode was not stable in the pocket environments of SV2B and SV2C (Figure S5). Especially in the SV2C system, **UCB-J** was found to be dissociating in the last 150 ns, and the ratio of water bridges in all types of interactions is the highest, because much more water molecules were present in the area close to upper lipid interface than in the buried initial binding site. Due to the high mobility of water molecules, such indirect contact is less stable than the direct hydrogen bond. Some geometrical conserved water molecules may be found (e.g., in over 50% of a simulation trajectory) around the hydrogen bonding distance of protein; however, no direct experimental evidence has been disclosed about the existence of functional water molecules in SV2 proteins. In SV2B, the π–π interactions were significantly present in 4 of the top-10 most interacted residues (Figure 4E), where W243 (ranked first), W607 (ranked second), and F220 (ranked sixth) are aligned to the W300, W666, and F277 residues in SV2A, respectively. The result demonstrates the importance of π–π interactions for SV2B to recognize **UCB-J**. Additionally, although the lysine residues mapping to K694 of SV2A were not forming direct hydrogen bonds with **UCB-J** in SV2B (K635) and SV2C (K697), in a large fraction of trajectories, this hydrogen bond was replaced by water bridges (Figure 4E,F,H,I). Therefore, the high selectivity of **UCB-J** for SV2A may be attributed to its unique cation−π and hydrogen bonding interactions with K694.

**Figure 5:**
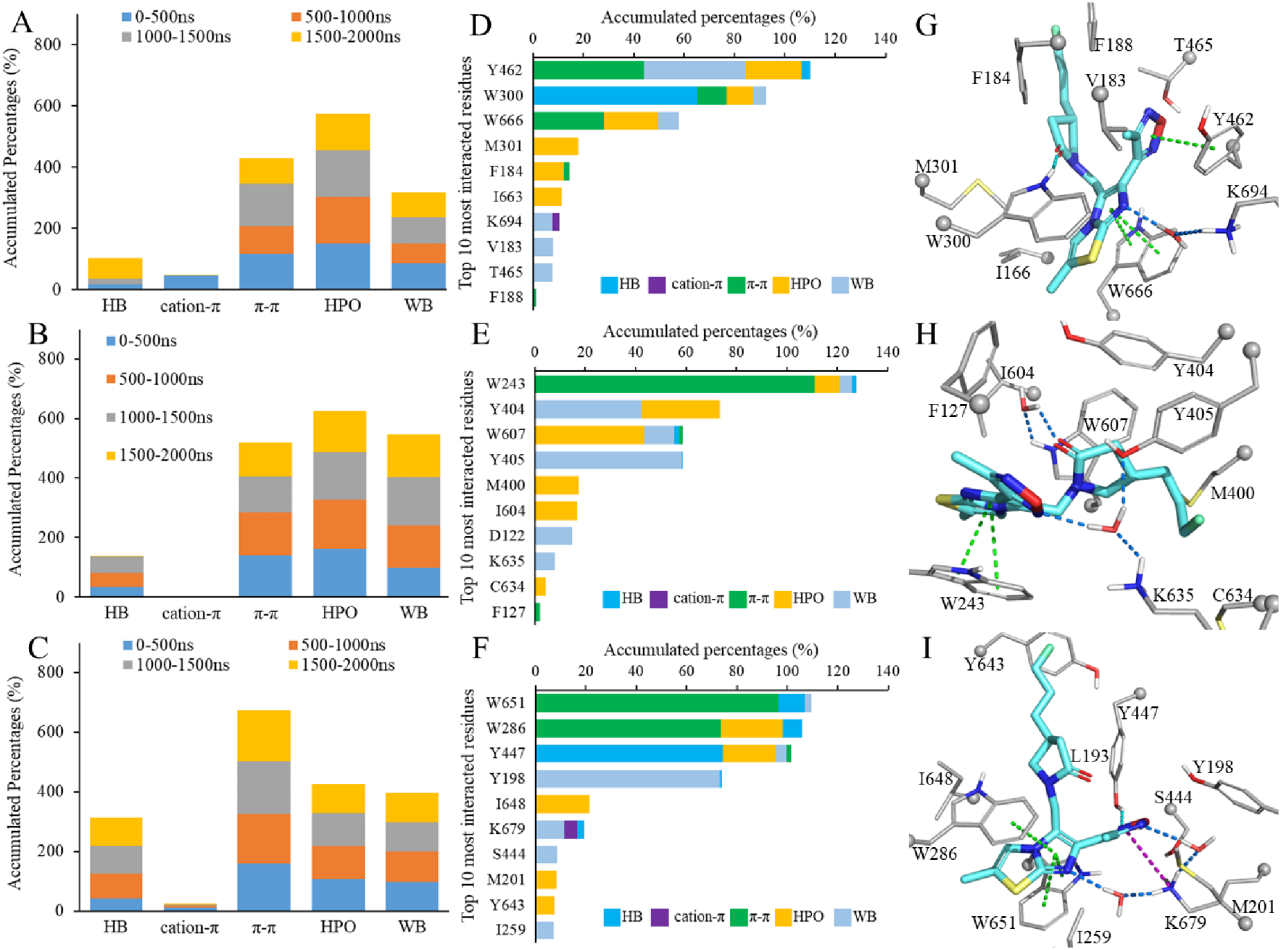
Simulation interaction diagrams for **UCB-F** in SV2A (subfigures A, D, and G in the first row), SV2B (B, E, and H in the second row), and SV2C (C, F, and I in the third row). Hydrogen bonding, nonspecific hydrophobic, and water bridge interactions are abbreviated as HB, HPO, and WB, respectively. The counting of hydrophobic interactions excludes the π–π and cation−π interactions. The top-10 most interacted residues in the trajectories of 1500–2000 ns are dissected in D–F. The representative snapshots are presented in G–I with the key residues, Cα atoms, and **UCB-F** colored in gray thin sticks, small spheres, and cyan sticks, respectively.

Although **UCB-F** has a larger aromatic scaffold, the cation−π interactions were only found in a small fraction of trajectories from 0 to 500 ns of SV2A (K694) and 1500–2000 ns of SV2C (K679) (Figure 5A–C). The number of hydrogen bonds increased in the last 500 ns simulation in the SV2A system (Figure 5A), which was contributed by W300 (Figure 5D). The existence of this hydrogen bond also decreased the number of π–π interactions between W300 and the imidazo[2,1-*b*]thiazol moiety of **UCB-F**. However, on the other side of the aromatic system of **UCB-F**, π–π interactions with Y462 and W666 were found to dominating the last 500 ns trajectory of SV2A. The Y462 is the top-1 most interacted residue in SV2A, but any two of the four types of interactions are often not coexisting in a single frame (Figure 5D,G), while in SV2A/**UCB-J** simulations, the cation−π and hydrogen bonding can exist simultaneously in a single snapshot (Figure 4G). This may also distinguish the difference in interactions between **UCB-J** and **UCB-F** with SV2A. Relatedly, the water bridges and nonspecific hydrophobic interactions in SV2B is the highest in all the three **UCB-F** simulations (Figure 4B). Hydrogen bonding between K635 (aligned to K694 in SV2A) and the 2-one oxygen of the pyrrolidone core almost vanished in the last 500 ns simulations (Figure 4B), which was replaced by a small portion of water bridge (Figure 4E,H). The π–π interactions with W243 (aligned to W300 in SV2A) might be the major driving force for recognizing **UCB-F** in SV2B. In the SV2C/**UCB-F** simulation, the top-3 most interacted residues, W651, W286, and W447 (mapped to W666, W300, and W461 in SV2A, respectively), are all with the accumulated ratio over 100% of the last 500 ns trajectory (Figure 5F), indicating more direct interactions in the SV2C/**UCB-F** system. The hydrogen bond interaction between **UCB-F** and SV2C increased after 500 ns, which was mainly contributed by Y447 that is aligned to Y461 in SV2A. Although in the docking pose, the 2-one oxygen of **UCB-F** is the hydrogen bond receptor, it was replaced by the N3 atom in the oxadiazol moiety in most snapshots of the trajectory from the last 500 ns (Figure 5I). Interestingly, no π–π interactions were found in the SV2C induced-fit docking (probably due to the large movement of aromatic residue to adopt **UCB-F**, Figure 3F), and the side chain atoms of W651 and W286 were found to quickly stack with the imidazo[2,1-*b*]thiazol moiety in the beginning of the MD simulation. Therefore, the sandwich-like π–π interactions with W651 and W286 may be the key for recognizing **UCB-F** in SV2C.

### Free Energy Profiles of UCB-J to SV2 from Different Trajectories

The analysis presented above qualifies the different preferences of interactions between **UCB-J** and **UCB-F** in SV2 proteins. We further performed MM/PBSA (The Molecular Mechanics Poisson–Boltzmann Surface Area) calculations for the 6 MD systems. From Table 1, the trend of binding free energies in each 500 ns period for **UCB-J** in SV2 protein are found to correlate well with that of the experimental p*K*_i_ values (8.15, 5.70, and 7.00 for SV2A, SV2B, and SV2C in mol, respectively).^24^ The binding affinities for **UCB-F** in SV2 proteins were predicted to follow the orders of SV2C > SV2B > SV2A. The free energies for **UCB-J** in SV2A drops about 5 kcal/mol after the first 500 ns simulation, which is caused by the fewer nonspecific hydrophobic interactions (Figure 4A), because the corresponding vdWs energy term is usually contributing the most to MM/PBSA binding free energies.^37,38^ However, this trend does not seem to work in SV2A/**UCB-F**, where the averaged PBSA energy increased gradually during the MD simulation, because the decreased nonspecific hydrophobic interaction was greatly compensated by the hydrogen bonding in the last 500 ns trajectory (Figure 5A). Although **UCB-J** was found to leave the initial binding site (where the key residues can be aligned to SV2A), the SV2C binding pocket is close to the upper lipid interface (Figure S4) still exhibits a better affinity to **UCB-J** than the SV2B pocket that is still aligned to the key residues in SV2A (Table 1).

**Table 1:** MM/PBSA Binding Free Energies (kcal/mol) for **UCB-J** and **UCB-F** in SV2 Proteins in Different Periods of Trajectories (500 ns Each, with 500 Frames)^a^

	time slots	UCB-J	UCB-F
SV2A	0–500 ns	–43.8 ± 0.7	–31.5 ± 3.1
	500–1000 ns	–36.0 ± 1.0	–33.8 ± 2.1
	1000–1500 ns	–36.7 ± 1.0	–36.1 ± 1.6
	1500–2000 ns	–38.1 ± 0.7	–38.4 ± 2.5
SV2B	0–500 ns	–17.9 ± 1.5	–36.3 ± 2.5
	500–1000 ns	–18.8 ± 1.1	–37.5 ± 2.1
	1000–1500 ns	–20.0 ± 1.4	–39.0 ± 2.5
	1500–2000 ns	–19.4 ± 1.4	–40.4 ± 2.1
SV2C	0–500 ns	–29.7 ± 1.0	–41.5 ± 2.1
	500–1000 ns	–25.6 ± 0.9	–45.6 ± 1.6
	1000–1500 ns	–25.4 ± 0.8	–46.2 ± 1.8
	1500–2000 ns	–22.4 ± 1.4	–45.9 ± 1.8

a The standard deviations with propagation of uncertainty formula are present here.

**Figure 6:**
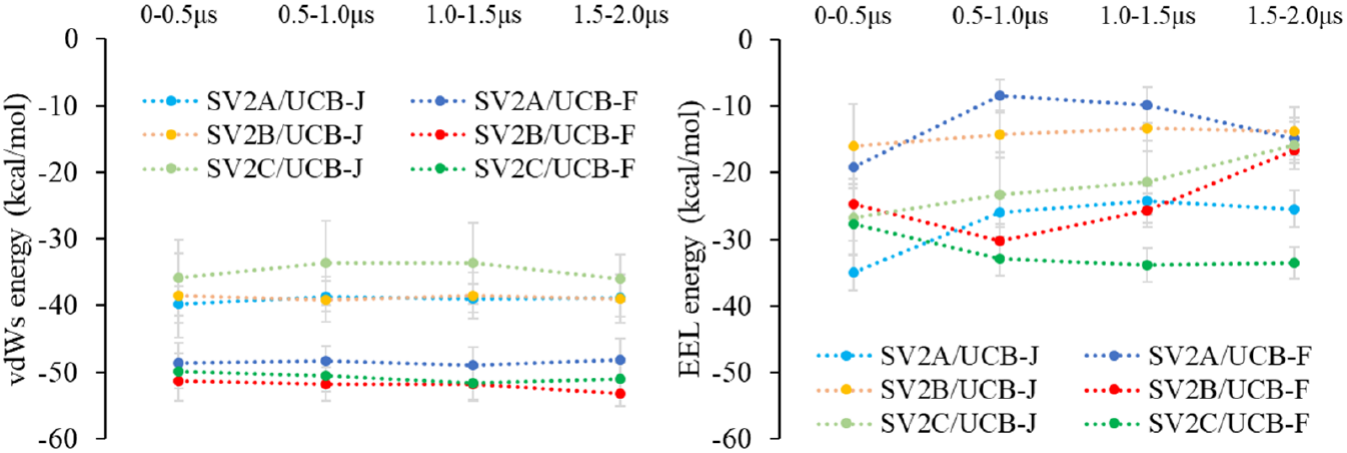
Van der Waals (vdWs) and Coulombic (EEL) energy terms for each system during the MD simulations. The standard deviation error bars are light gray.

We further analyzed the two major energy terms, the van der Waals (vdWs) and the Coulombic (EEL), which contribute to the total PBSA free energy in each stage of the MD simulations. By using the *eneopt = 2* option, both the van der Waals and Coulombic energies are computed via the summation of pairwise atomic interactions. The sum of the two terms is named binding free energy in the gas phase.^37^ From Figure 6, it is clear that the vdWs is relative to the size of ligand when they bind to the buried site of a protein, in which the larger size **UCB-F** contributes more due to more contacts with SV2 proteins. The vdWs term is the major contributing term to the final PBSA free energies (Table S1), which is also correlated with the numbers of hydrophobic interactions (π–π, cation−π, and other nonspecific interactions). For the EEL term, its values can be affected by the number of hydrogen bond and salt bridge interactions. More cation−π interactions might also contribute to the more negative EEL energy, resulting in a better predicted PBSA free energy for **UCB-J** in SV2A (Figure 6). It is also worth to note that **UCB-F** in SV2C exhibits a stronger EEL energy than **UCB-J** in SV2A after 500 ns, which might be caused by the more hydrogen bonding interactions in SV2C/**UCB-F** (Figure 4C).

For the nonpolar solvation term, the value is typically small and shows a minor variation among similar ligands.^38^ In our case, even though the structure of UCB-J is not so similar to **UCB-F**, similar nonpolar solvation energies were observed (Table S1). The polar solvation energy term is obtained here by solving the PB equation numerically. A significant positive value of this term suggests a transition of a charged/polar molecule (ligand) from a hydrophilic environment (solvent) to the hydrophobic environment (the enclosed active site of the protein). The variations of this term do not show significant differences across the various systems as indicated in Table S1, generally displaying a larger EPB for **UCB-F** compared to **UCB-J**.

## Conclusion

This study aimed to unravel the key structural details in the binding of two distinct ligands, **UCB-J** and **UCB-F**, which may be relatively selective to SV2A and SV2C, respectively.

The binding patterns of **UCB-J** and **UCB-F** to the three proteins SV2A, SV2B, and SV2C were all explored by docking and relative large-scale MD simulations with respect to the various binding patterns, types of interactions, and binding free energies. The interaction patterns comprised hydrogen bonding and nonspecific hydrophobic interactions, as well as water bridge, π–π, and cation−π interactions. The docking derived binding modes showed a strong preference for hydrogen bonding of **UCB-J** and **UCB-F** with only the lysine residue in the pocket (K694, K635, and K679 for SV2A, SV2B, and SV2C, respectively). However, the MD simulations indicated that only **UCB-J** in SV2A maintains this hydrogen bond with additional cation−π interactions with the pyridine moiety of **UCB-J**, which can explain why **UCB-J** is selective SV2A tracer due to such a unique interaction fingerprint. For **UCB-F** in SV2 proteins, the interaction pattern shows that there are more hydrogen bonding and π–π interactions maintained in the trajectories, which might also explain its selectivity over SV2C. The MM/PBSA binding free energies can further explain the trend of selectivity of **UCB-J** over SV2A and **UCB-F** over SV2C. One example is that although **UCB-J** in SV2B also exhibits significant π–π interactions they are much weaker than those exhibited by **UCB-F** in SV2C as they are not sustained in a face-to-face manner. The latter configuration gives optimal π–π interaction that can be found in the MD simulation for the SV2C/**UCB-F** complex. Overall, we show in this work that a molecular detail analysis of the binding between SV2 proteins and tracers offers a possibility to understand the binding in terms of particular chemical interactions, and to provide useful insight into future development of new selective tracers targeting SV2A, SV2B, or SV2C.

## Methods

### Preparation of the RosettaFold Predicted Structures

All the initial predicted structures were prepared using the CHARMM-GUI web server.^39,40^ The general CHARMM force field and CHARMM36m force field were applied for the ligands and protein atoms, respectively.^41−43^ A total of 256 POPC (1-palmitoyl-2-oleoyl-glycero-3-phosphocholine) lipid molecules were added to each system with the 1:1 ratio on the *x* and *y* directions of the orthorhombic box, which has the size of 101.2, 101.2, and 143.4 Å on the *x*, *y*, and *z* directions, respectively. The thickness of water on the top and bottom areas of the membrane was set to 17.5, resulting in ∼31300 water molecules added to each system. Counter ions were added to neutralize the system followed by more sodium and chloride ions being added to reach the ion strength of 0.1 M.

### Induced-Fit Docking

The structures of the **UCB-J** and **UCB-F** were manually depicted in the Maestro interface of Schrödinger Suite (version 2021-4),^44^ followed by the preparation with the LigPrep module for the assignment of atom types, bond orders, and atomic partial charges.^45^ The active sites for the induced-fit docking workflow, which implements the Glide (for docking) and Prime (for binding site residue energy minimization) modules,^46−48^ were defined by the geometrical center of W300/W243/W286, W666/W607/W651, and Y462/Y405/Y448 for SV2*A*/2B/2C. For the initial docking attempts, the van der Waals scaling factors for receptor and ligand were both assigned to 0.50 with the side chain atoms of W666/W607/W651 and Y462/Y405/Y448 trimmed. The grid size/length for adopting the ligand was set to 30 Å. The side chain atoms of the residues within 5.0 Å of the ligand poses obtained from the first attempt of docking were energy-minimized by the prime module, of which the top-20 best energy-ranked receptor models within 30 kcal/mol were chosen for the redocking of ligands using the standard precision mode.

### Molecular Dynamics Simulations

All the simulations were performed using GROMACS version 2023.2.^49,50^ CHARMM36 (version Jul.2022) and the general charm force field (CGenFF) were used for the protein and ligand atoms, respectively. The TIP3P water model was used for the solvent molecules.^51^ The steepest descent method was used for minimizing the system, followed by heating to 300 K using the *v-rescale* temperature coupling scheme^52^ and maintaining the pressure to 1 atm using the Parrinello–Rahman pressure coupling scheme^53^ for the 100 ps equilibrations under the NVT and NPT ensembles, respectively. The LINCS constrain was applied for all the bonds involving hydrogen atoms,^54^ with the nonboned interactions within 12 Å being considered. The Particle Mesh Ewald method was used to recover the long-range electrostatic interaction with 1.0-Å grid spacing.^55^ The trajectories were saved every 200 ps with a time step of 2 fs and a random initial velocity for each particle. The trajectories were inspected by the Visual Molecular Dynamics (VMD, version 1.9.4a57).^56^ The simulation interaction diagram workflow implemented in Desmond (version 2021-4)^57^ was used to dissect the various interactions between ligands and SV2 protein as well as identifying the top-10 most contacted residues in each system.

### Binding Free Energy Calculations

To qualify the selectivity profile of **UCB-J** and **UCB-F** against SV2A, SV2B, and SV2C, we use the gmx_MMPBSA toolkit (version 1.6.2)^58^ to calculate the MM/PBSA (Molecular Mechanics/Poisson–Boltzmann Surface Area) free energies for each system at each stage with the interval of 500 ns.^37^ This toolkit transfers gromacs trajectory and CHARMM force field into Amber formats and uses the *mmpbsa_py_energy*/*sander* module of AMBER automatically for the PBSA calculations. A special *charmm_radii* set (*PBRadii* = 7)^59^ were used for the calculation of snapshots extracted from 0 to 500, 500–1000, 1000–1500, and 1500–2000 ns (1 frame/ns). The ionic strength was set to 0.15 M (*istrng* = 0.15). The other parameters were left as default as implemented in gmx_MMPBSA.

## Supporting Information Available

Supplementary text regarding the criteria in simulation interaction analysis, Supporting Table 1 of the energy terms in MM/PBSA calculations, and Supporting Figures 1 to 5: Sequences alignment of human SV2 proteins; superimposition of the top-5 predicted structures for SV2 proteins; time course for the RMSD values during the MD simulations; comparison of ligand conformations before and after MD simulations; and hydrogen bonding analysis for 2 μs MD simulations of **UCB-J** in SV2 protein (PDF)

## References

[ref1] Buckley K.; Kelly R. B. Identification of a Transmembrane Glycoprotein Specific for Secretory Vesicles of Neural and Endocrine Cells. J. Cell Biol. 1985, 100, 1284–1294. 10.1083/jcb.100.4.1284. 2579958 PMC2113776

[ref2] Bajjalieh S. M.; Peterson K.; Shinghal R.; Scheller R. H. SV2, a Brain Synaptic Vesicle Protein Homologous to Bacterial Transporters. Science 1992, 257, 1271–1273. 10.1126/science.1519064. 1519064

[ref3] Feany M. B.; Lee S.; Edwards R. H.; Buckley K. M. The Synaptic Vesicle Protein SV2 is a Novel Type of Transmembrane Transporter. Cell 1992, 70, 861–867. 10.1016/0092-8674(92)90319-8. 1355409

[ref4] Bartholome O.; Van den Ackerveken P.; Sánchez Gil J.; de la Brassinne Bonardeaux O.; Leprince P.; Franzen R.; Rogister B. Puzzling Out Synaptic Vesicle 2 Family Members Functions. Front. Mol. Neurosci. 2017, 10, 148 10.3389/fnmol.2017.00148. 28588450 PMC5438990

[ref5] Stout K. A.; Dunn A. R.; Hoffman C.; Miller G. W. The Synaptic Vesicle Glycoprotein 2: Structure, Function, and Disease Relevance. ACS Chem. Neurosci. 2019, 10, 3927–3938. 10.1021/acschemneuro.9b00351. 31394034 PMC11562936

[ref6] Appelbaum L. G.; Shenasa M. A.; Stolz L.; Daskalakis Z. Synaptic Plasticity and Mental Health: Methods, Challenges and Opportunities. Neuropsychopharmacology 2023, 48, 113–120. 10.1038/s41386-022-01370-w. 35810199 PMC9700665

[ref7] Pao S. S.; Paulsen I. T.; Saier M. H. Major Facilitator Superfamily. Microbiol. Mol. Biol. Rev. 1998, 62, 1–34. 10.1128/MMBR.62.1.1-34.1998. 9529885 PMC98904

[ref8] Hediger M. A.; Clémençon B.; Burrier R. E.; Bruford E. A. The ABCs of Membrane Transporters in Health and Disease (SLC series): Introduction. Mol. Aspects Med. 2013, 34, 95–107. 10.1016/j.mam.2012.12.009. 23506860 PMC3853582

[ref9] Bajjalieh S. M.; Peterson K.; Linial M.; Scheller R. H. Brain contains two forms of synaptic vesicle protein 2. Proc. Natl. Acad. Sci. U. S. A. 1993, 90, 2150–2154. 10.1073/pnas.90.6.2150. 7681585 PMC46043

[ref10] Bajjalieh S.; Frantz G.; Weimann J.; McConnell S.; Scheller R. Differential Expression of Synaptic Vesicle Protein 2 (SV2) Isoforms. J. Neurosci. 1994, 14, 5223–5235. 10.1523/JNEUROSCI.14-09-05223.1994. 8083732 PMC6577109

[ref11] Janz R.; Südhof T. C. SV2C is a Synaptic Vesicle Protein with an Unusually Restricted: ocalization: Anatomy of a Synaptic Vesicle Protein Family. Neuroscience 1999, 94, 1279–1290. 10.1016/S0306-4522(99)00370-X. 10625067

[ref12] Crowder K. M.; Gunther J. M.; Jones T. A.; Hale B. D.; Zhang H. Z.; Peterson M. R.; Scheller R. H.; Chavkin C.; Bajjalieh S. M. Abnormal Neurotransmission in Mice Lacking Synaptic Vesicle Protein 2A (SV2A). Proc. Natl. Acad. Sci. U. S. A. 1999, 96, 15268–15273. 10.1073/pnas.96.26.15268. 10611374 PMC24809

[ref13] Mendoza-Torreblanca J. G.; Vanoye-Carlo A.; Phillips-Farfán B. V.; Carmona-Aparicio L.; Gómez-Lira G. Synaptic Vesicle Protein 2A: Basic Facts and Role in Synaptic Function. Eur. J. Neurosci. 2013, 38, 3529–3539. 10.1111/ejn.12360. 24102679

[ref14] Peng L.; Tepp W. H.; Johnson E. A.; Dong M. Botulinum Neurotoxin D Uses Synaptic Vesicle Protein SV2 and Gangliosides as Receptors. PLoS Pathog. 2011, 7, e1002008 10.1371/journal.ppat.1002008. 21483489 PMC3068998

[ref15] Benoit R. M.; Frey D.; Hilbert M.; Kevenaar J. T.; Wieser M. M.; Stirnimann C. U.; McMillan D.; Ceska T.; Lebon F.; Jaussi R. Structural Basis for Recognition of Synaptic Vesicle Protein 2C by Botulinum Neurotoxin A. Nature 2014, 505, 108–111. 10.1038/nature12732. 24240280

[ref16] Yao G.; Zhang S.; Mahrhold S.; Lam K.-H.; Stern D.; Bagramyan K.; Perry K.; Kalkum M.; Rummel A.; Dong M. N-linked Glycosylation of SV2 is Required for Binding and Uptake of Botulinum Neurotoxin A. Nat. Struct. Mol. Biol. 2016, 23, 656–662. 10.1038/nsmb.3245. 27294781 PMC5033645

[ref17] Cai H.; Mangner T. J.; Muzik O.; Wang M.-W.; Chugani D. C.; Chugani H. T. Radiosynthesis of ^11^C-Levetiracetam: A Potential Marker for PET Imaging of SV2A Expression. ACS Med. Chem. Lett. 2014, 5, 1152–1155. 10.1021/ml500285t. 25313330 PMC4190623

[ref18] Finnema S. J.; Nabulsi N. B.; Eid T.; Detyniecki K.; Lin S.-F.; Chen M.-K.; Dhaher R.; Matuskey D.; Baum E.; Holden D.; Spencer D. D.; Mercier J.; Hannestad J.; Huang Y.; Carson R. E. Imaging Synaptic Density in the Living Human Brain. Sci. Transl. Med. 2016, 8 (348), ra348396–ra348396. 10.1126/scitranslmed.aaf6667. 27440727

[ref19] Serrano M. E.; Becker G.; Bahri M. A.; Seret A.; Mestdagh N.; Mercier J.; Mievis F.; Giacomelli F.; Lemaire C.; Salmon E. Evaluating the *in vivo* Specificity of [^18^F]UCB-H for the SV2A Protein, Compared with SV2B and SV2C in Rats Using microPET. Molecules 2019, 24, 1705 10.3390/molecules24091705. 31052478 PMC6538996

[ref20] Becker G.; Dammicco S.; Bahri M. A.; Salmon E. The Rise of Synaptic Density PET Imaging. Molecules 2020, 25, 2303 10.3390/molecules25102303. 32422902 PMC7288098

[ref21] Carson R. E.; Naganawa M.; Toyonaga T.; Koohsari S.; Yang Y.; Chen M.-K.; Matuskey D.; Finnema S. J. Imaging of Synaptic Density in Neurodegenerative Disorders. J. Nucl. Med. 2022, 63, 60S–67S. 10.2967/jnumed.121.263201. 35649655 PMC12530506

[ref22] Lynch B. A.; Lambeng N.; Nocka K.; Kensel-Hammes P.; Bajjalieh S. M.; Matagne A.; Fuks B. The Synaptic Vesicle Protein SV2A is the Binding Site for the Antiepileptic Drug Levetiracetam. Proc. Natl. Acad. Sci. U. S. A. 2004, 101, 9861–9866. 10.1073/pnas.0308208101. 15210974 PMC470764

[ref23] Gillard M.; Fuks B.; Leclercq K.; Matagne A. Binding Characteristics of Brivaracetam, a Selective, High Affinity SV2A Ligand in Rat, Mouse and Human Brain: Relationship to anti-Convulsant Properties. Eur. J. Pharmacol. 2011, 664, 36–44. 10.1016/j.ejphar.2011.04.064. 21575627

[ref24] Nabulsi N. B.; Mercier J.; Holden D.; Carré S.; Najafzadeh S.; Vandergeten M.-C.; Lin S.-F.; Deo A.; Price N.; Wood M. Synthesis and Preclinical Evaluation of ^11^C-UCB-J as a PET Tracer for Imaging the Synaptic Vesicle Glycoprotein 2A in the Brain. J. Nucl. Med. 2016, 57, 777–784. 10.2967/jnumed.115.168179. 26848175

[ref25] Mercier J.; Provins L.; Valade A. Discovery and Development of SV2A PET Tracers: Potential for Imaging Synaptic Density and Clinical Applications. Drug Discovery Today: Technol. 2017, 25, 45–52. 10.1016/j.ddtec.2017.11.003. 29233267

[ref26] Li S.; Cai Z.; Zhang W.; Holden D.; Lin S.-F.; Finnema S. J.; Shirali A.; Ropchan J.; Carre S.; Mercier J. Synthesis and *in vivo* Evaluation of [^18^F]UCB-J for PET Imaging of Synaptic Vesicle Glycoprotein 2A (SV2A). Eur. J. Nucl. Med. Mol. Imaging 2019, 46, 1952–1965. 10.1007/s00259-019-04357-w. 31175396 PMC6810698

[ref27] Warnock G. I.; Aerts J.; Bahri M. A.; Bretin F.; Lemaire C.; Giacomelli F.; Mievis F.; Mestdagh N.; Buchanan T.; Valade A. Evaluation of a New [^18^F] Labeled Tracer Targeting Synaptic Vesicle Protein 2C by ex vivo Autoradiography and in vivo PET Study in Rat Brain. J. Nucl. Med. 2014, 55, 1336–1341. 10.2967/jnumed.113.136143. 24935992

[ref28] Dunn A. R.; Stout K. A.; Ozawa M.; Lohr K. M.; Hoffman C. A.; Bernstein A. I.; Li Y.; Wang M.; Sgobio C.; Sastry N.; Cai H.; Caudle W. M.; Miller G. W. Synaptic Vesicle Glycoprotein 2C (SV2C) Modulates Dopamine Release and is Disrupted in Parkinson Disease. Proc. Natl. Acad. Sci. U. S. A. 2017, 114 (11), E2253–E2262 10.1073/pnas.1616892114. 28246328 PMC5358362

[ref29] Rai S. N.; Birla H.; Zahra W.; Sen Singh S.; Singh S. P. Commentary: Synaptic vesicle Glycoprotein 2C (SV2C) Modulates Dopamine Release and is Disrupted in Parkinson Disease. Front. Synaptic Neurosci. 2018, 9, 18 10.3389/fnsyn.2017.00018. 29354047 PMC5758556

[ref30] Dunn A. R.; Hoffman C. A.; Stout K. A.; Ozawa M.; Dhamsania R. K.; Miller G. W. Immunochemical Analysis of the Expression of SV2C in Mouse, Macaque andHuman Brain. Brain Res. 2019, 1702, 85–95. 10.1016/j.brainres.2017.12.029. 29274878 PMC6013333

[ref31] Tan Z.; Lin Y.; Zhou M.; Guo W.; Qiu J.; Ding L.; Wu Z.; Xu P.; Chen X. Correlation of SV2C rs1423099 Single Nucleotide Polymorphism with Sporadic Parkinson’s Disease in Han Population in Southern China. Neurosci. Lett. 2023, 813, 137426 10.1016/j.neulet.2023.137426. 37544580

[ref32] Shi J.; Anderson D.; Lynch B. A.; Castaigne J.-G.; Foerch P.; Lebon F. Combining Modelling and Mutagenesis Studies of Synaptic Vesicle Protein 2A to Identify a Series of Residues Involved in Racetam Binding. Biochem. Soc. Trans. 2011, 39 (5), 1341–1347. 10.1042/BST0391341. 21936812

[ref33] Lee J.; Daniels V.; Sands Z. A.; Lebon F.; Shi J.; Biggin P. C. Exploring the Interaction of SV2A with Racetams Using Homology Modelling, Molecular Dynamics and Site-Directed Mutagenesis. PLoS One 2015, 10, e0116589 10.1371/journal.pone.0116589. 25692762 PMC4333566

[ref34] Baek M.; DiMaio F.; Anishchenko I.; Dauparas J.; Ovchinnikov S.; Lee G. R.; Wang J.; Cong Q.; Kinch L. N.; Schaeffer R. D. Accurate Prediction of Protein Structures and Interactions Using a Three-track Neural Network. Science 2021, 373, 871–876. 10.1126/science.abj8754. 34282049 PMC7612213

[ref35] Omasits U.; Ahrens C. H.; Müller S.; Wollscheid B. Protter: Interactive Protein Feature Visualization and Integration with Experimental Proteomic Data. Bioinformatics 2014, 30, 884–886. 10.1093/bioinformatics/btt607. 24162465

[ref36] Jumper J.; Evans R.; Pritzel A.; Green T.; Figurnov M.; Ronneberger O.; Tunyasuvunakool K.; Bates R.; Žídek A.; Potapenko A. Highly Accurate Protein Structure Prediction with AlphaFold. Nature 2021, 596, 583–589. 10.1038/s41586-021-03819-2. 34265844 PMC8371605

[ref37] Kollman P. A.; Massova I.; Reyes C.; Kuhn B.; Huo S.; Chong L.; Lee M.; Lee T.; Duan Y.; Wang W. Calculating Structures and Free Energies of Complex Molecules: Combining Molecular Mechanics and Continuum Models. Acc. Chem. Res. 2000, 33, 889–897. 10.1021/ar000033j. 11123888

[ref38] Genheden S.; Ryde U. The MM/PBSA and MM/GBSA Methods to Estimate Ligand-binding Affinities. Expert Opin. Drug Discovery 2015, 10, 449–461. 10.1517/17460441.2015.1032936. PMC4487606 25835573

[ref39] Jo S.; Kim T.; Iyer V. G.; Im W. CHARMM-GUI: A Web-based Graphical User Interface for CHARMM. J. Comput. Chem. 2008, 29, 1859–1865. 10.1002/jcc.20945. 18351591

[ref40] Lee J.; Cheng X.; Swails J. M.; Yeom M. S.; Eastman P. K.; Lemkul J. A.; Wei S.; Buckner J.; Jeong J. C.; Qi Y. CHARMM-GUI Input Generator for NAMD, GROMACS, AMBER, OpenMM, and CHARMM/OpenMM Simulations Using the CHARMM36 Additive Force Field. J. Chem. Theory Comput. 2016, 12, 405–413. 10.1021/acs.jctc.5b00935. 26631602 PMC4712441

[ref41] Vanommeslaeghe K.; MacKerell A. D. Automation of the CHARMM General Force Field (CGenFF) I: Bond Perception and Atom Typing. J. Chem. Inf. Model. 2012, 52, 3144–3154. 10.1021/ci300363c. 23146088 PMC3528824

[ref42] Vanommeslaeghe K.; Raman E. P.; MacKerell A. D. Automation of the CHARMM General Force Field (CGenFF) II: Assignment of Bonded Parameters and Partial Atomic Charges. J. Chem. Inf. Model. 2012, 52, 3155–3168. 10.1021/ci3003649. 23145473 PMC3528813

[ref43] Huang J.; MacKerell A. D. CHARMM36 all-atom additive protein force field: Validation based on comparison to NMR data. J. Comput. Chem. 2013, 34, 2135–2145. 10.1002/jcc.23354. 23832629 PMC3800559

[ref44] Schrödinger Schrödinger Release 2021–4; Schrödinger, LLC: New York, NY, 2021.

[ref45] Schrödinger LigPrep; Schrödinger LLC: New York, NY, 2021.

[ref46] Schrödinger Prime; Schrödinger LLC: New York, NY, 2021.

[ref47] Friesner R. A.; Banks J. L.; Murphy R. B.; Halgren T. A.; Klicic J. J.; Mainz D. T.; Repasky M. P.; Knoll E. H.; Shelley M.; Perry J. K.; et al. Glide: A New Approach for Rapid, Accurate Docking and Scoring. 1. Method and Assessment of Docking Accuracy. J. Med. Chem. 2004, 47, 1739–1749. 10.1021/jm0306430. 15027865

[ref48] Halgren T. A.; Murphy R. B.; Friesner R. A.; Beard H. S.; Frye L. L.; Pollard W. T.; Banks J. L. Glide: A New Approach for Rapid, Accurate Docking and Scoring. 2. Enrichment Factors in Database Screening. J. Med. Chem. 2004, 47, 1750–1759. 10.1021/jm030644s. 15027866

[ref49] Berendsen H. J. C.; van der Spoel D.; van Drunen R. GROMACS: A Message-Passing Parallel Molecular Dynamics Implementation. Comput. Phys. Commun. 1995, 91, 43–56. 10.1016/0010-4655(95)00042-E.

[ref50] Abraham M. J.; Murtola T.; Schulz R.; Páll S.; Smith J. C.; Hess B.; Lindahl E. GROMACS: High Performance Molecular Simulations through Multi-level Parallelism from Laptops to Supercomputers. SoftwareX 2015, 1, 19–25. 10.1016/j.softx.2015.06.001.

[ref51] Jorgensen W. L.; Chandrasekhar J.; Madura J. D.; Impey R. W.; Klein M. L. Comparison of Simple Potential Functions for Simulating Liquid Water. J. Chem. Phys. 1983, 79, 926–935. 10.1063/1.445869.

[ref52] Bussi G.; Donadio D.; Parrinello M. Canonical Sampling Through Velocity Rescaling. J. Chem. Phys. 2007, 126 (1), 014101 10.1063/1.2408420. 17212484

[ref53] Parrinello M.; Rahman A. Crystal Structure and Pair Potentials: A Molecular-Dynamics Study. Phys. Rev. Lett. 1980, 45, 1196–1199. 10.1103/PhysRevLett.45.1196.

[ref54] Hess B.; Bekker H.; Berendsen H. J. C.; Fraaije J. G. E. M. LINCS: A Linear Constraint Solver for Molecular Simulations. J. Comput. Chem. 1997, 18, 1463–1472. 10.1002/(SICI)1096-987X(199709)18:12<1463:AID-JCC4>3.0.CO;2-H.

[ref55] Darden T.; York D.; Pedersen L. Particle Mesh Ewald: An N·log(N) Method for Ewald Sums in Large Systems. J. Chem. Phys. 1993, 98, 10089–10092. 10.1063/1.464397.

[ref56] Humphrey W.; Dalke A.; Schulten K. VMD: Visual Molecular Dynamics. J. Mol. Graph. 1996, 14, 33–38. 10.1016/0263-7855(96)00018-5. 8744570

[ref57] Bowers K. J.; Chow E.; Xu H.; Dror R. O.; Eastwood M. P.; Gregersen B. A.; Klepeis J. L.; Kolossvary I.; Moraes M. A.; Sacerdoti F. D., Proceedings of the 2006 ACM/IEEE conference on Supercomputing; Association for Computing Machinery: Tampa, FL, 2006; p 746.

[ref58] Valdés-Tresanco M. S.; Valdés-Tresanco M. E.; Valiente P. A.; Moreno E. gmx_MMPBSA: A New Tool to Perform End-State Free Energy Calculations with GROMACS. J. Chem. Theory Comput. 2021, 17, 6281–6291. 10.1021/acs.jctc.1c00645. 34586825

[ref59] Nina M.; Beglov D.; Roux B. Atomic Radii for Continuum Electrostatics Calculations Based on Molecular Dynamics Free Energy Simulations. J. Phys. Chem. B 1997, 101, 5239–5248. 10.1021/jp970736r.

