# Supplementary material for: Exploring the Interactions between two Ligands, UCB-J and UCB-F, and Synaptic Vesicle Glycoprotein 2 Isoforms
Source: ACS Chem Neurosci. 2024 May 3;15(10):2018–27. doi: 10.1021/acschemneuro.4c00029 (PMC11099911; doi:10.1021/acschemneuro.4c00029)
Supplement: Supplementary file 1 — cn4c00029_si_001.pdf [file cn4c00029_si_001.pdf]

Supporting Information

For

Exploring the interactions between two ligands, UCB-J and UCB-F, and synaptic vesicle glycoprotein 2 isoforms

Junhao Li[a*], Rongfeng Zou[a], Andrea Varrone[b], Sangram Nag[b], Christer Halldin[b], Hans Ågren[a*]

[a] *Department of Physics and Astronomy, Uppsala University, Box 516, SE-751 20 Uppsala, Sweden*
[b] *Department of Clinical Neuroscience, Karolinska Institutet, Karolinska University Hospital Solna, SE-171 76 Stockholm, Sweden*

Correspondence: Junhao Li, junhao.li@physics.uu.se; Hans Ågren, hans.agren@physics.uu.se;

Supplementary text

The five categories of protein-ligand interactions/contacts exhibit on Figures 4 and 5 are defined by a series of geometrical criteria (adopted from the simulation interaction report generated by the Desmond analysis toolkit)

Hydrogen Bonds (HB) usually contribute significantly to the binding of ligand to the active site of protein. The current geometric criteria for protein-ligand HB are defined as the distance of 2.5 Å between the donor and acceptor atoms (D—H···A), a donor angle greater than 120° between the donor-hydrogen-acceptor atoms (D—H···A), and an acceptor angle greater than 90° between the hydrogen-acceptor-bonded_atom atoms (H···A—X).

Hydrophobic interactions are important for the *van der* Waals energy and fall into three subtypes (as presented in Figures 4 and 5), which are **cation-π**, **π- π**, and **non-specific hydrophobic** (**HPO**) interactions. The current geometric criteria for hydrophobic interactions are as follows:

cation-π: Aromatic and charged groups within 4.5 Å;

π-π: Two aromatic groups stacked face-to-face or face-to-edge;

HPO: A non-specific hydrophobic sidechain within 3.6 Å of a ligand's aromatic or aliphatic carbons.

Water Bridges (WB) are hydrogen-bonded protein-ligand interactions mediated by a water molecule. The hydrogen-bond geometry is slightly relaxed from the standard H-bond definition.

The current geometric criteria for a protein-water or water-ligand H-bond are: a distance of 2.8 Å between the donor and acceptor atoms (D—H···A); a donor angle of ≥110° between the donor-hydrogen-acceptor atoms (D—H···A); and an acceptor angle of ≥90° between the hydrogen-acceptor-bonded_atom atoms (H···A—X).

Table S1. Energy terms of all the MM/PBSA calculations (kcal/mol)

Systems	Time courses	Statistics	vdWs	EEL	EPB	ENPOLAR
SV2A/ UCB-J	0.0-0.5μs	Average	-39.85	-34.99	34.84	-3.83
		SD	2.70	6.36	4.42	0.11
		SEM	0.12	0.28	0.20	0.00
	0.5-1.0μs	Average	-38.61	-25.99	32.65	-4.09
		SD	2.25	7.21	4.51	0.15
		SEM	0.10	0.32	0.20	0.01
	1.0-1.5μs	Average	-38.93	-24.17	30.54	-4.12
		SD	2.18	7.75	3.86	0.15
		SEM	0.10	0.35	0.17	0.01
	1.5-2.0μs	Average	-38.83	-25.43	30.21	-4.10
		SD	2.81	7.42	4.42	0.17
		SEM	0.13	0.33	0.20	0.01
SV2B/ UCB-J	0.0-0.5μs	Average	-38.48	-16.01	40.82	-4.27
		SD	2.43	6.35	9.67	0.14
		SEM	0.11	0.28	0.43	0.01
	0.5-1.0μs	Average	-39.11	-14.33	38.97	-4.29
		SD	2.03	3.37	6.84	0.09
		SEM	0.09	0.15	0.31	0.00
	1.0-1.5μs	Average	-38.58	-13.25	36.07	-4.28
		SD	2.13	3.48	6.70	0.10
		SEM	0.10	0.16	0.30	0.00
	1.5-2.0μs	Average	-39.01	-13.78	37.66	-4.26
		SD	1.98	3.65	7.10	0.10
		SEM	0.09	0.16	0.32	0.00
SV2C/ UCB-J	0.0-0.5μs	Average	-35.81	-26.71	36.96	-4.13
		SD	2.30	5.74	4.83	0.12
		SEM	0.10	0.26	0.22	0.01
	0.5-1.0μs	Average	-33.6	-23.26	35.55	-4.26
		SD	2.31	6.35	6.00	0.13
		SEM	0.10	0.28	0.27	0.01
	1.0-1.5μs	Average	-33.67	-21.36	33.86	-4.27
		SD	2.46	6.12	5.01	0.14
		SEM	0.11	0.27	0.22	0.01
	1.5-2.0μs	Average	-35.95	-15.87	33.69	-4.26
		SD	2.73	3.54	4.95	0.12
		SEM	0.12	0.16	0.22	0.01
SV2A/ UCB-F	0.0-0.5μs	Average	-48.65	-19.18	41.07	-4.74
		SD	3.08	10.2	11.85	0.13
		SEM	0.14	0.46	0.53	0.01

	0.5-1.0μs	Average	-48.33	-8.36	27.71	-4.77
		SD	2.30	2.62	4.45	0.11
		SEM	0.10	0.12	0.20	0.01
	1.0-1.5μs	Average	-48.95	-9.85	27.47	-4.74
		SD	2.74	3.13	4.24	0.11
		SEM	0.12	0.14	0.19	0.00
	1.5-2.0μs	Average	-48.19	-14.91	29.35	-4.62
		SD	3.18	3.46	4.02	0.12
		SEM	0.14	0.15	0.18	0.01
SV2B/ UCB-F	0.0-0.5μs	Average	-51.32	-24.64	44.35	-4.66
		SD	3.01	10.15	13.02	0.10
		SEM	0.13	0.45	0.58	0.00
	0.5-1.0μs	Average	-51.82	-30.27	49.21	-4.66
		SD	2.52	5.62	6.62	0.08
		SEM	0.11	0.25	0.30	0.00
	1.0-1.5μs	Average	-51.72	-25.6	43.06	-4.7
		SD	2.54	10.86	12.42	0.09
		SEM	0.11	0.49	0.56	0.00
	1.5-2.0μs	Average	-53.19	-16.7	34.22	-4.70
		SD	1.89	4.69	6.30	0.08
		SEM	0.08	0.21	0.28	0.00
SV2C/ UCB-F	0.0-0.5μs	Average	-49.83	-27.72	40.82	-4.76
		SD	2.56	5.41	4.89	0.11
		SEM	0.11	0.24	0.22	0.01
	0.5-1.0μs	Average	-50.47	-32.99	42.53	-4.66
		SD	2.51	4.79	3.82	0.10
		SEM	0.11	0.21	0.17	0.00
	1.0-1.5μs	Average	-51.69	-33.86	43.95	-4.65
		SD	2.47	4.23	3.60	0.08
		SEM	0.11	0.19	0.16	0.00
	1.5-2.0μs	Average	-51.02	-33.49	43.28	-4.65
		SD	2.35	4.47	3.64	0.09
		SEM	0.11	0.20	0.16	0.00

The total binding free energy is the sum of van der waals (vdWs), electrostatic (EEL), polar solvation (EPB), and non-polar solvation (ENPOLAR) energy terms, in which the gas phase binding free energy and the implicit solvation energy are calculated from vdWs+EEL and EPB+ENPOLAR, respectively.

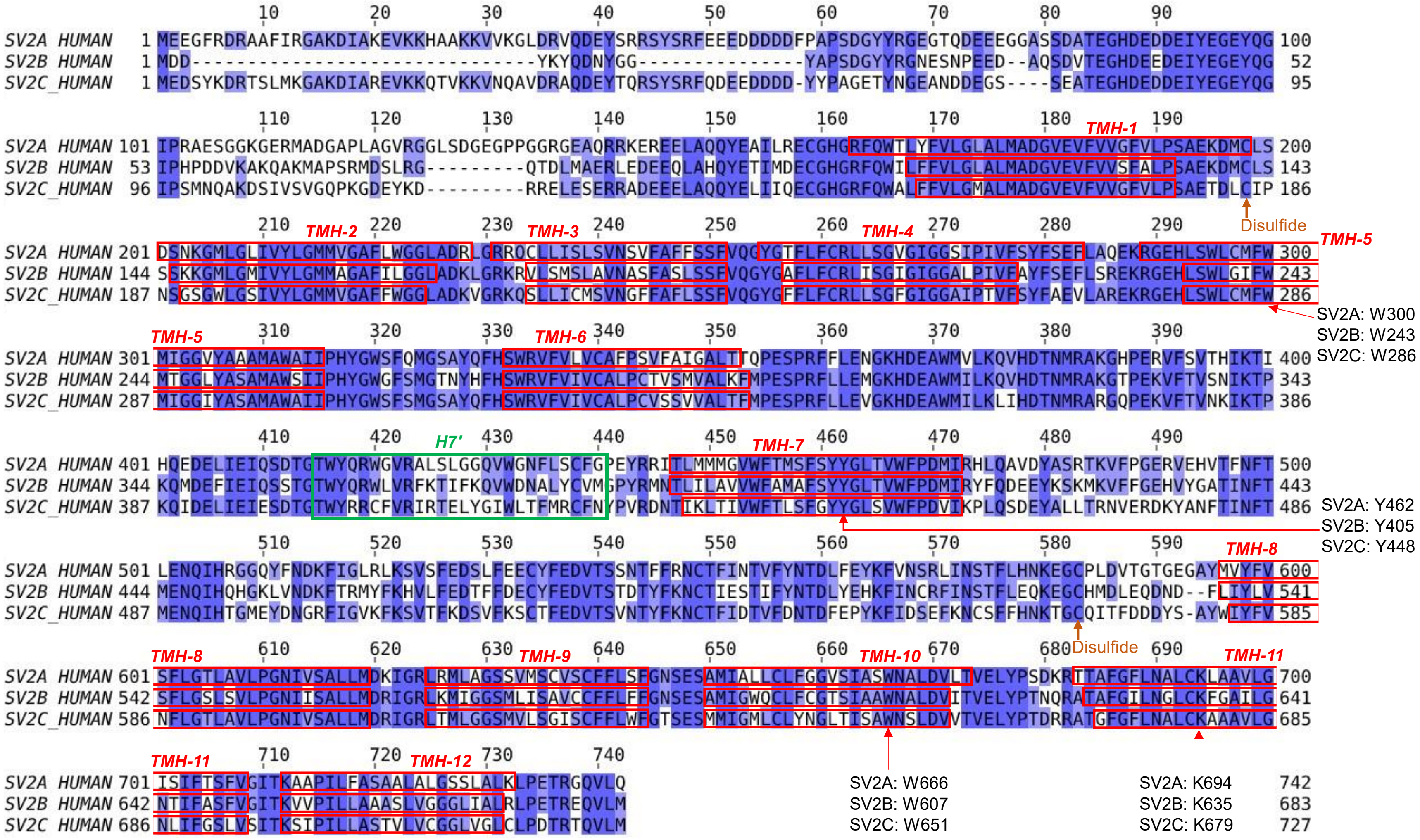

Figure S1. Alignment of the sequences of human SV2 proteins.

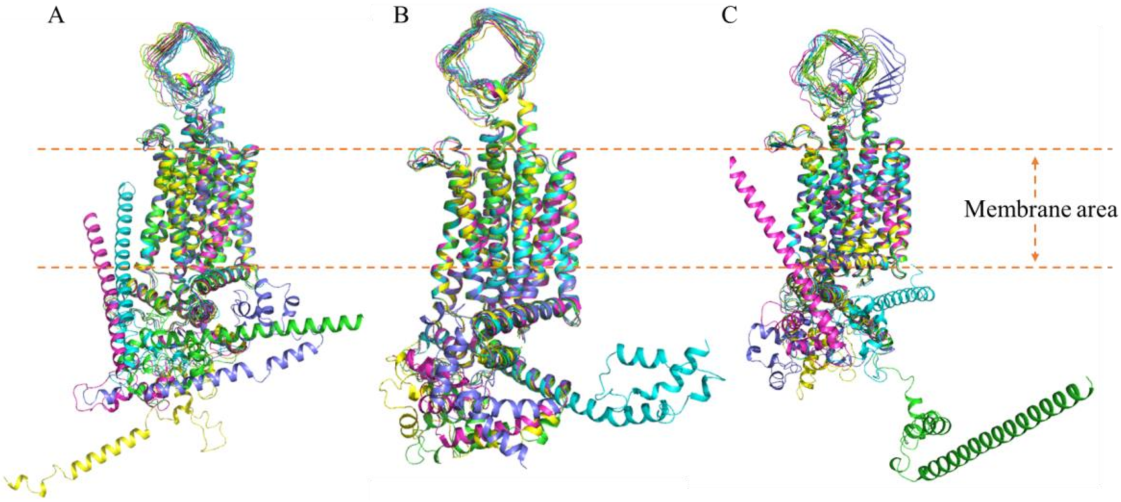

Figure S2. Superimposing the models generated from the RosettaFold server. A). SV2A models; B). SV2B models; C). SV2C models

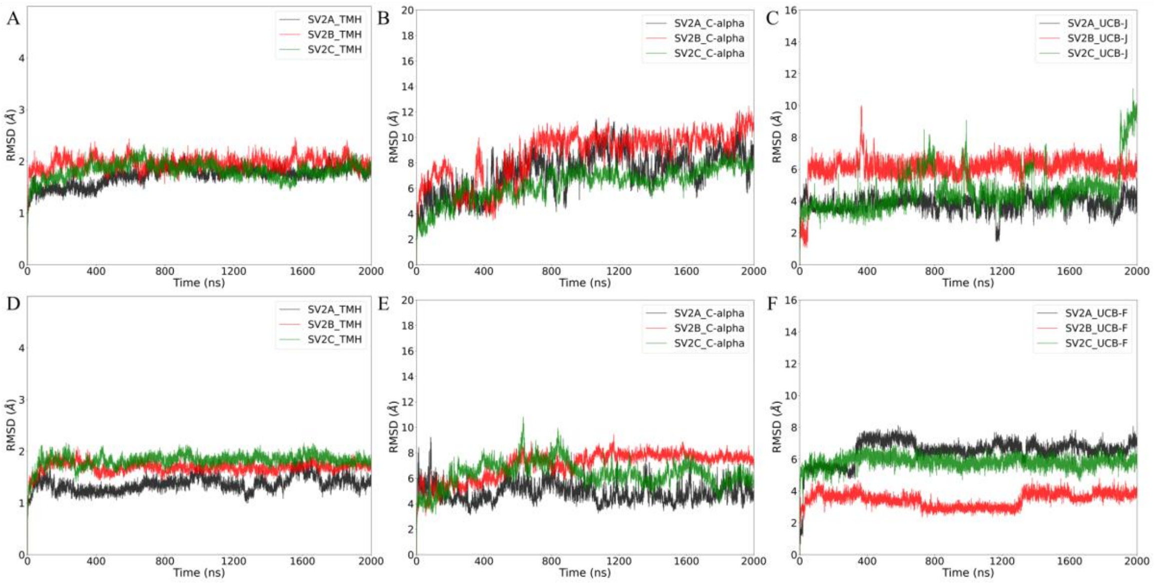

Figure S3. Time course of root meas square deviation (RMSD) for the simulations with UCB-J (A, B, and C) and UCB-F (D, E, and F). The RMSD values for the Cα atoms of transmembrane helices (TMH), all the Cα atoms, and the non-hydrogen atoms of ligands are presented in the left, middle, and third columns, respectively.

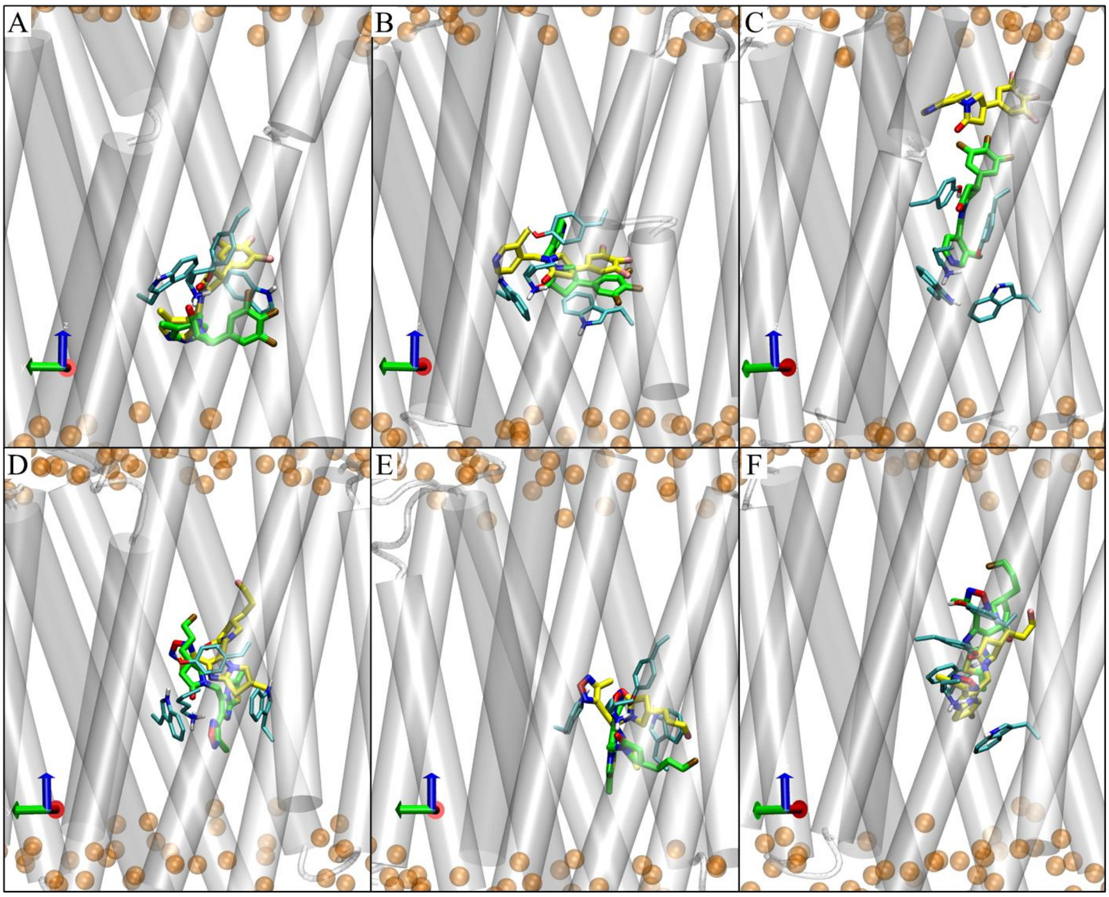

Figure S4. Superimposing the first and last snapshots of **UCB-J** (A, B, and C) and **UCB-F** (D, E, and F) in the binding pockets of SV2A (A and D, i.e. the first column), SV2B (B and E, i.e. the second column), and SV2C (C and F, i.e. the third column). Ligands from the first and last snapshot are depicted as green and yellow sticks, respectively. The important residues and tansmembrane helices are depicted as thin cyan sticks and transparent tubes, respectively.

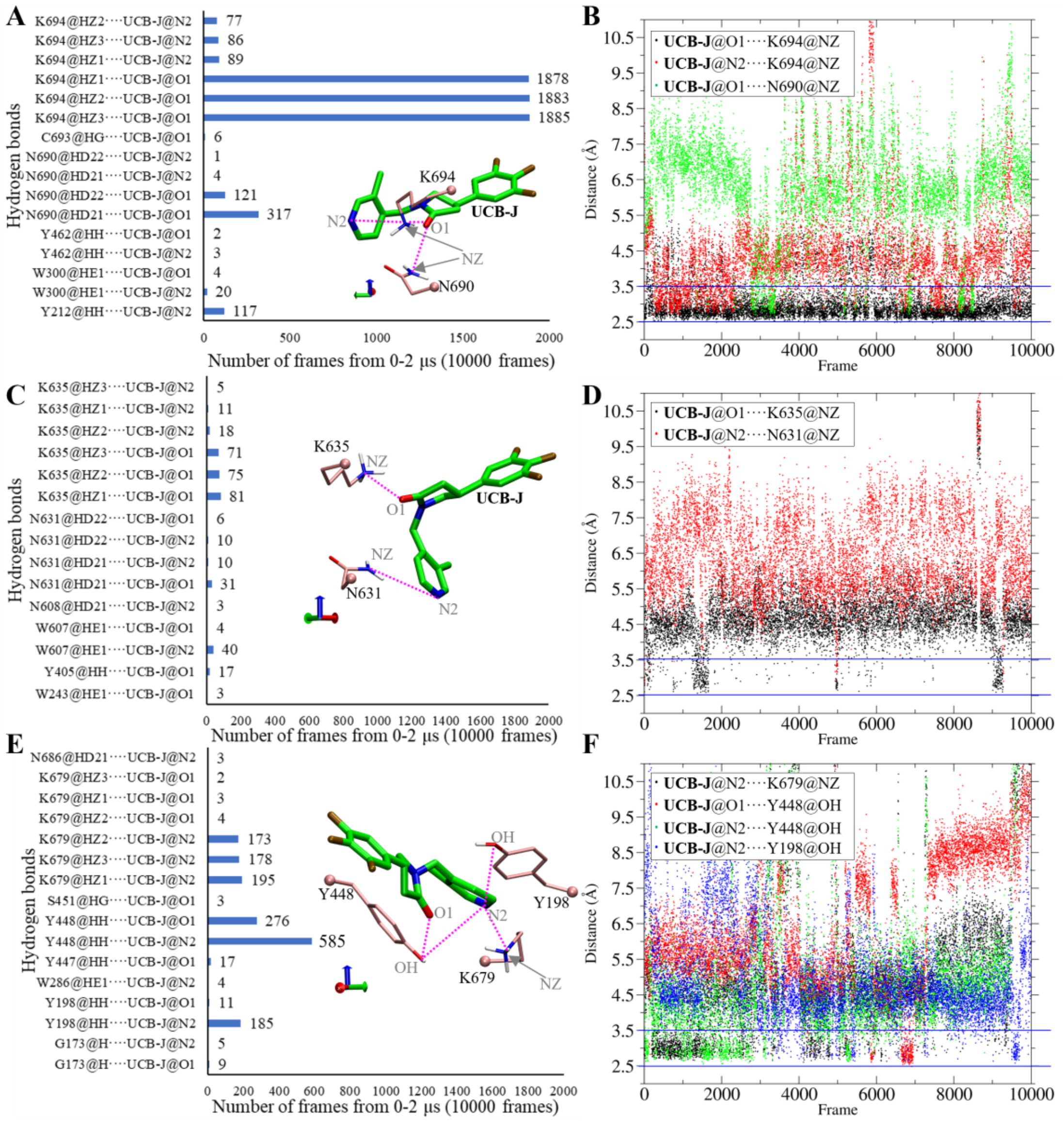

Figure S5. Hydrogen bonding analysis for **UCB-J** in SV2A (A and B), SV2B (C and D), and SV2C (E and F). The left column (A, C, and E) shows the number of frames (from 0-2 μs, 10000 frames) with the criteria for a hydrogen bond between UCB-J and protein being fulfilled. The right column (B, D, and F) shows the time course of the heavy atom distance of the major hydrogen bonds found in the left column. The positions of the related heavy atoms are depicted in the left column for each of the SV2 proteins.
